# Estimation and mapping of soil texture content based on unmanned aerial vehicle hyperspectral imaging

**DOI:** 10.1038/s41598-023-40384-2

**Published:** 2023-08-29

**Authors:** Qi Song, Xiaohong Gao, Yuting Song, Qiaoli Li, Zhen Chen, Runxiang Li, Hao Zhang, Sangjie Cai

**Affiliations:** 1 School of Geographical Sciences, Qinghai Normal University, Xining, 810008 China; 2 Qinghai Province Key Laboratory of Physical Geography and Environmental Process, Xining, 810008 China; 3 Ministry of Education Key Laboratory of Tibetan Plateau Land Surface Processes and Ecological Ministry of Education Key Laboratory of Tibetan Plateau Land Surface Processes and Ecological Conservation, Xining, 810008 China; 4 Academy of Plateau Science and Sustainability, Xining, 810008 China

**Keywords:** Environmental impact, Geophysics

## Abstract

Soil texture is one of the important physical and natural properties of soil. Much of the current research focuses on soil texture monitoring using non-imaging geophysical spectrometers. However there are fewer studies utilizing unmanned aerial vehicle (UAV) hyperspectral data for soil texture monitoring. UAV mounted hyperspectral cameras can be used for quickly and accurately obtaining high-resolution spatial information of soil texture. A foundation has been laid for the realization of rapid soil texture surveys using unmanned airborne hyperspectral data without field sampling. This study selected three typical farmland areas in Huangshui Basin of Qinghai as the study area, and a total of 296 soil samples were collected. Data calibration of UAV spectra using laboratory spectra and field in situ spectra to explore the feasibility of applying laboratory soil texture models directly to field conditions. This results show that UAV hyperspectral imagery combined with machine learning can obtain a set of ideal processing methods. The pre-processing of the spectral data can obtain high accuracy of soil texture estimation and good mapping effect. The results of this study can provide effective technical support and decision-making assistance for future agricultural land planning on the Tibetan Plateau. The main innovation of this study is to establish a set of processing procedures and methods applicable to UAV hyperspectral imagery to provide data reference for monitoring soil texture in agricultural fields on the Tibetan Plateau.

## Introduction

Soil texture is an important physical and natural property of soil. It represents the combination of soil particles and is closely related to soil ventilation permeability, and water and fertility retention. According to the soil particle size, soil texture can be divided into clay particles (< 0.002 mm), silt particles (0.002–0.05 mm), and sand particles (0.05–2 mm)^1,2^. Soil texture significantly affects the soil bacterial community structure and diversity, therefore, affecting soil fertility^3^. It is an important index in the field of cultivated land quality and in the evaluation of crop suitability^4^. The estimation and mapping of the spatial distribution of soil texture can not only enrich and improve the soil digital database, but also provide a basis and data support for research on the spatial distribution of soil attributes and agricultural production planning^5^. Therefore, precision agricultural management is urgently required for the high-resolution and high-precision quantification and monitoring of the spatial and temporal distribution characteristics of soil texture at the field scale.

Traditional soil texture measurement methods rely on field soil sampling and laboratory chemical analysis, which are time-consuming, laborious, and costly, making it difficult to conduct large scale and multifrequency soil texture content monitoring^6,7^. In recent years, rapidly developing vision-near-infrared hyperspectral technology has been widely used in soil texture content estimation to address the contradiction between the demand for big data of soil texture and high cost^8^. Depending on the spectral response relationship between soil spectral reflectance and soil texture, many researchers have used ground object spectrometry to develop soil hyperspectral technology as a conventional means of quantifying soil texture^9–11^. However, soil texture inversion based on a ground-object spectrometer usually obtains spotlike data with low density. This makes it difficult to meet the requirements of rapid visualization of spatial distribution in the context of precision agriculture^12^. UAV platforms have the advantages of mobility and flexibility and have been widely used in land resource space surveys in recent years^13^. Through the organic combination of UAV and hyperspectral technology, vegetation growth monitoring, accurate classification and ground object identification, pest monitoring, production and yield estimation, and other diverse applications have been conducted. However, relatively few applications have used this technology to monitor soil properties, particularly soil texture^14^.

Most of the current research on mapping soil texture content focuses on field field sampling and collecting soil spectral information using non-imaging spectrometers^15^. Soil texture content mapping was eventually carried out using interpolation. With the development of UAV hyperspectral technology, it is able to provide high spatial resolution and high spectral resolution imagery, from which more accurate soil texture content mapping results can be obtained^16^. Therefore, this study will use UAV hyperspectral imagery for soil texture content mapping.

As emerging tools, UAV have the advantages of portability, high spatial resolution, high flexibility, independent selection of flight time, and the ability to carry a variety of spectral cameras^17^. This can quickly and efficiently achieve remote sensing image acquisition in a specified area^18^. A UAV hyperspectral system is an effective tool for monitoring and mapping the spatial distribution of soil textures. The hyperspectral camera carried by UAV can not only obtain ultra-high spatial resolution images, but also centimeter-level remote sensing images of farmland rapidly and in real time^19^. This means that they can be used to effectively assist agricultural operators in operation management and regulation^20^. Selige et al.^21^ used airborne hyperspectral images to study the spatial variability of soil texture and organic matter in the field and found that soil organic matter and soil texture were related to the spectral characteristics of the airborne spectrograph. The modeling accuracy R^2^ (R-squared) reached 0.9. Aldana-Jague et al.^22^ used multi-spectral cameras carried by a small UAV and combined them with a structure-from-motion algorithm to test the SOC inversion of farmland bare soil at the Lausanne experimental station in England. The organic combination of UAV platforms and soil spectral data has broad prospects in soil organic carbon prediction. Ge et al.^23^ used UAV hyperspectral images and combined a pre-processed spectral index with the Extreme learning machine (ELM) algorithm to obtain a high-precision soil moisture content estimation model at the regional scale (R^2^ = 0.907). Low-altitude UAV equipped with hyperspectral cameras for image acquisition can easily obtain high spatial resolution multiband from visible light to near infrared remote sensing data to achieve a balance between cost and availability. Currently, the use of UAV remote sensing images to study field soil texture content is relatively low.

The acquisition of UAV hyperspectral data in the field environment is affected by soil moisture. Soil moisture has been identified as one of the main reasons for the decline in accuracy of soil attribute estimations^24^. For the spectral estimation of soil texture, given that a change in moisture affects the change in soil texture content, it has a greater impact on the spectrum^25,26^. Therefore, it is necessary to study the spectral effects of soil moisture and its removal methods. Several methods have been proposed to improve the prediction accuracy of soil properties under field conditions and overcome the influence of soil moisture on the prediction of soil texture using visible and near-infrared spectroscopy. Ji et al.^27^ respectively conducted field in situ and indoor spectral measurements of paddy soil in Zhejiang Province. The accuracy R^2^ and recurrent pattern detection (RPD) of field in situ partial least-squares regression (PLSR) model corrected by the direct spectral (DS) conversion method increased from 0.25 to 0.69 and from 0.35 to 1.61. The results have shown that DS could effectively eliminate the influence of water and environmental factors on the soil spectrum and improve the prediction accuracy of soil texture. Hu et al.^28^ applied direct and piecewise direct standardization, general least-squares weighting, and orthogonal signal correction to eliminate soil water from hyperspectral fields. The results have shown that the correction results of direct standardization were the most accurate. Minasny et al.^29^ studied the effect of eliminating a certain amount of soil moisture on the hyperspectral estimation of organic carbon using a laboratory water design for soils in southern New South Wales, Australia. Dor et al.^30^ standardized and corrected soil spectral data obtained under different soil water environments using an internal soil standard in Australia, thereby improving the comparability and transferability of various spectral data. However, at present, the university method is predominantly applied to field spectral data obtained based on the Analytical Spectral Devices (ASD) series ground object spectrometer. To date, there has been no research on correcting the spectral data obtained by the UAV high-resolution spectrometer. Studies on the influence of soil moisture on the spectral estimation of soil attribute contents have been mainly based on the water test design of indoor air-dried soil samples. Controlling the soil moisture content lacks the real situation of undisturbed soil and in-depth analysis, and the practicability and applicability of elimination methods are limited. Therefore, it is necessary to study the removal of soil moisture factors and quantitatively detect soil texture. Solving the problem of moisture affecting the inversion accuracy of soil texture is also helpful for the collaborative operation of spectrometers and agricultural machinery to realize the online detection of soil texture content by intelligent agricultural machinery.

Therefore, based on the image data obtained by the hyperspectral sensor mounted on the UAV platform, this study tested the applicability of high-resolution spectral data provided by the UAV to invert the soil texture content in three typical farmland areas of the Huangshui Watershed in Qinghai Province. UAV data were corrected by calibrating laboratory and field in situ data. Realizing the initial exploration from laboratory soil texture spectral inversion modeling to field applications. Establishment of a rapid, accurate and detailed technical system for assessing spatial and temporal changes in soil texture on agricultural land.

Research innovation: (1) establish a set of processing procedures and methods applicable to UAV hyperspectral images; (2) evaluate the effectiveness of different spectral pre-processing methods in extracting suitable feature information for soil texture inversion; (3) analyze the spatial distribution characteristics of soil texture estimated by different machine learning algorithms, with a view to providing data references for the monitoring of soil texture content in farmland and the development of agroecological balance in the Qinghai-Tibetan Plateau. development.

## Materials and methods

### Study area

**Figure 1:**
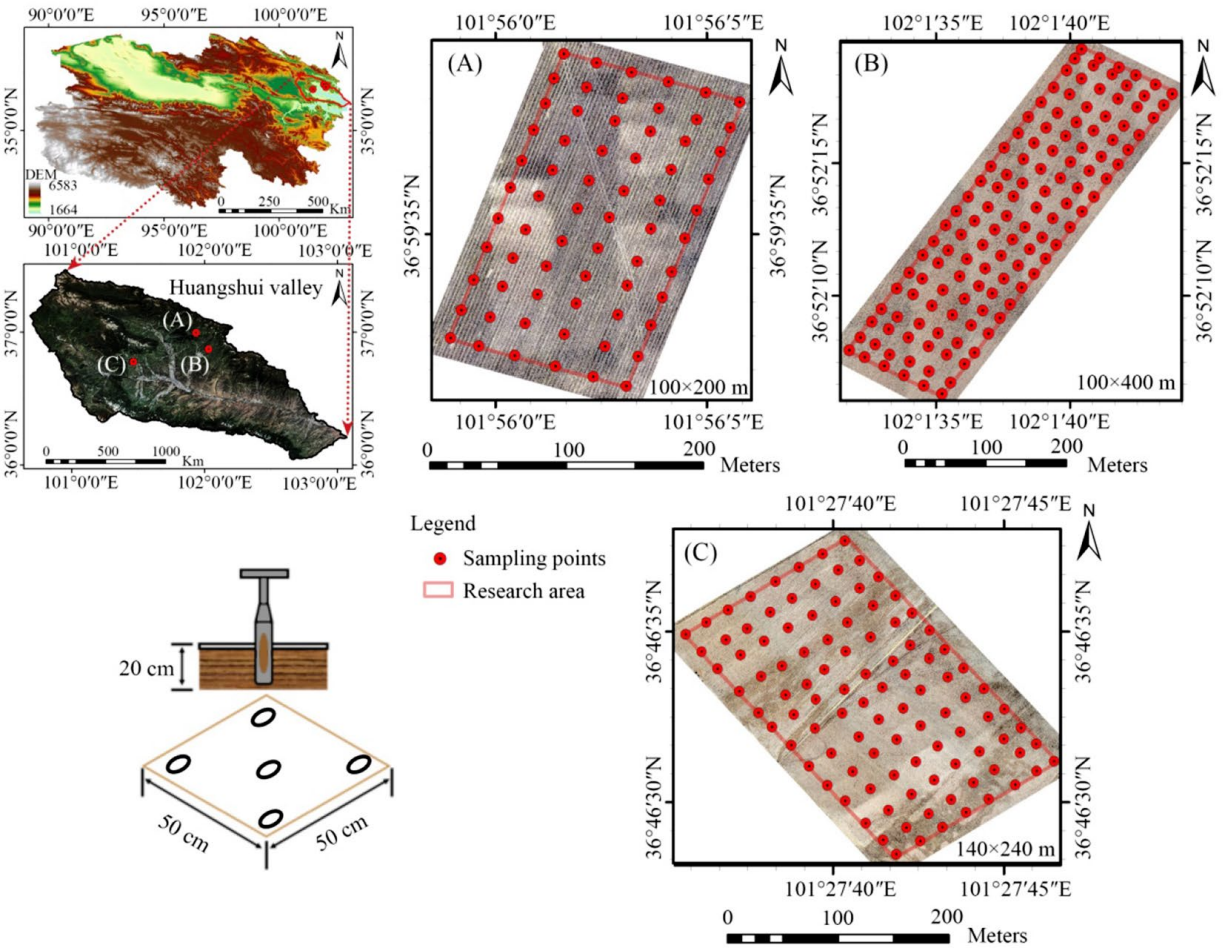
Distribution map of sampling points.

The study area was located in Huangshui Watershed, Qinghai Province, China (36° 02′–37° 28′ N, 100° 42′–103° 04′ E; Fig. 1), which forms part of the transition zone from the Loess Plateau to the Qinghai–Tibet Plateau. The overall terrain is high in the northwest, low in the southeast, long from east to west, and narrow from north to south. The altitude ranged between 1655 and 4860 m. The soil types were chestnut, chernozem, calcium ash, and alpine meadow soils. The plateau climate was an arid, semi-arid continental climate, with a long sunshine time, and nocturnal and diurnal temperature differences. Three farmlands in the Huangshui Watershed were selected as research objects. Farmland A, with an area of 2 ha (200 m × 100 m), was located in the Nanmen Gorge of Huzhu, China, and the soil type was chernozem. The farmland area was located in Zhuozhatan, Huzhu, with an area of 4 ha (400 × 100 m), and the soil type was chernozem. The farmland area was located in Huangzhong, with an area of 3.36 ha (240 × 140 m), and the soil type was chestnut.

### Soil samples and field in situ spectral collection

**Figure 2:**
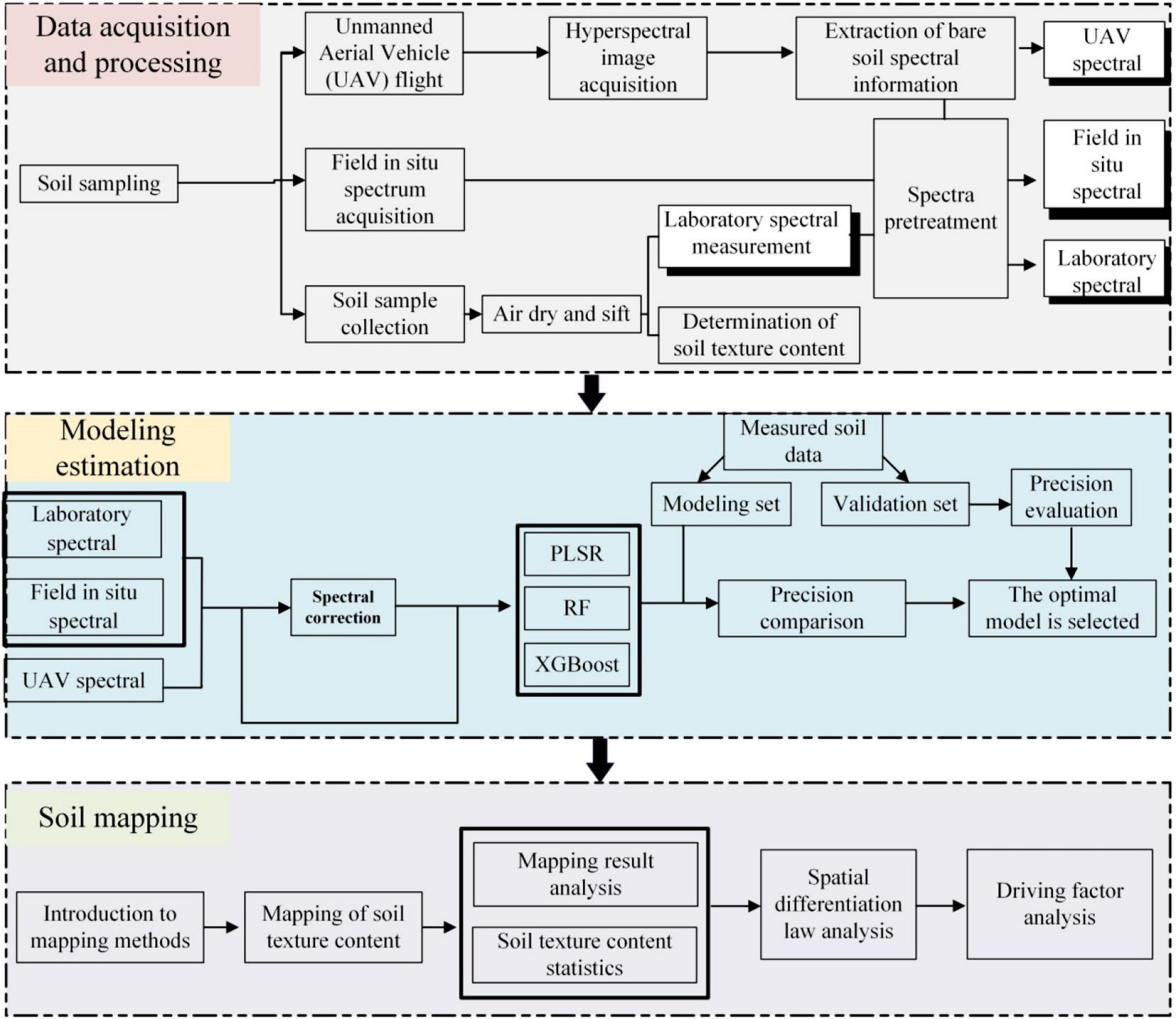
Technique flowchart.

The technical flowchart of this study is shown in Fig. 2. A total of 66, 126, and 104 soil samples were collected from Farmland areas A, B, and C on September 21, 2021, March 23–25, 2022, and March 27–28, 2022, respectively (Fig. 1). The surface 0–20 cm of the soil was collected using a five-point sampling method, and the soil samples were successively packed into sealed bags and numbered. An *American FieldSpec4* ground feature spectrometer (350–2500 nm) was used for the field spectral acquisition. The acquisition time was 11:00–15:30 under cloudless skies with winds less than Level 3. Before spectrum collection, the debris on the soil surface was cleaned, the soil surface was organized and levelled, and the optical fiber handle was vertically placed approximately 15 cm from the ground. Five spectra were collected from the four directions of east, west, north, and south for each sample point, and the arithmetic mean of the 20 spectra was used as the field in-situ spectral data for the sample point.

### UAV image acquisition and preprocessing

**Figure 3:**
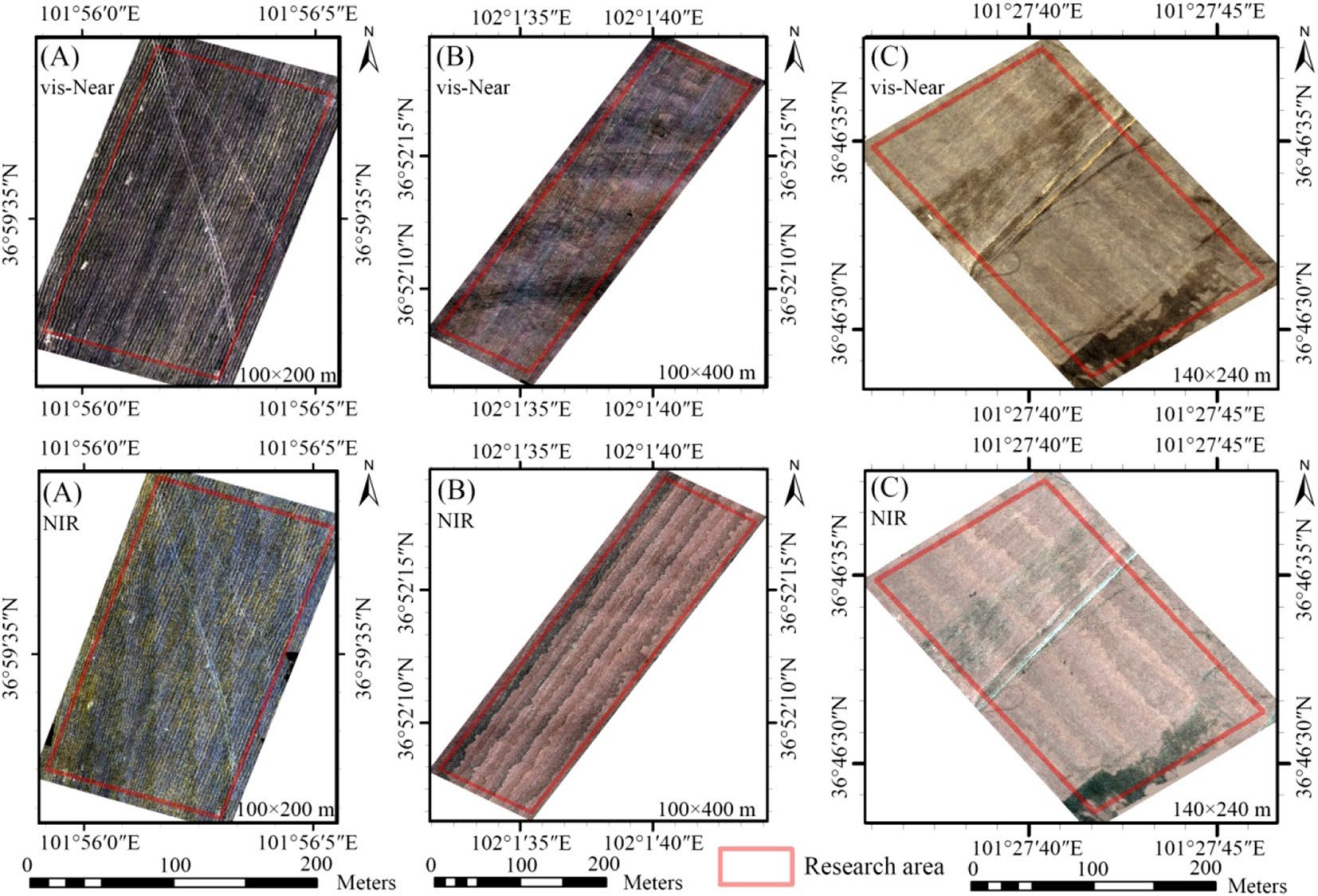
Hyperspectral cameras, field work and preprocessed hyperspectral images.

Using *DJI's M600 Pro UAV* as a platform, the *Resonon Pika L vis-Near* (400–1000 nm model: *Resonon Pika L*) and NIR (Near Infrared Spectrum 900–1700 nm model: *Pika NIR-640 Resonon PIKA Us*) hyperspectral camera (Fig. 3). On September 21, 2021, March 23, 2022, and March 27, 2022, hyperspectral images were captured in flight in farmland areas A, B, and C, respectively. The flight time was 11:00–15:30, the weather was clear and cloudless, the wind was less than three degrees, the flight altitude was 150 m, and the speed was 4.3 m/s.

For pre-processing, the flight path of the UAV was viewed in *Sbgcenter* software and the flight path data was exported. Then, *Omap* software was used to segment the boundary of the exported flight path data and remove invalid routes. *Airline Division* software was used to pre-cut the hyperspectral image according to the airline conditions, and a complete hyperspectral image was obtained. Radiometric calibration and atmospheric correction were conducted on the cut image using *MegaCube* software. The corrected image was then imported into *ArcGIS* software for geographic registration. The reference data were the remote procedure call file delivered by the UAV and the orthographic base map taken. After registration, the images were spliced using *ENVI* software. *MegaCube* software was used to create an image hypercube, which was then converted into a hyperspectral image. Therefore, pre-processed vis-Near and NIR images were obtained (Fig. 3). The coordinates of the actual sampling points were imported into the hyperspectral image to obtain the UAV spectral reflectance data of all sample points, and noisy bands (975–1033 nm and 1323–1479 nm) were removed.

### Soil sample determination and spectrum pretreatment

Soil samples are naturally air-dried in the laboratory, removed from impurities and then ground and screened. Weigh 0.5 g sample into a small beaker, add 10 ml 10% H_2_O_2_, heat and stir to react to remove organic matter. Then 10 ml 10% HCl was added, heated and stirred to remove carbonates. Remove the beaker and let it cool for 24 h. Remove the supernatant and add an appropriate amount of (NaPO_3_)_6_. Stir well and use Master sizer 2000 laser particle size analyzer to determine the texture content of the sample.

American FieldSpec4 ground feature spectrometer (350–2500 nm) was used for laboratory spectrum acquisition. The soil sample was placed in a black container with a diameter of 10 cm and a depth of 1.5 cm. The supporting high-density reflection probe was used to measure the spectrum closely to the soil sample. 20 spectra were measured at each sample point.

Since the wavelength range and spectral resolution of soil spectral data measured by ASD (Analytical Spectral Devices) FieldSpec 4 ground object spectrometer were different from the hyperspectral data of UAV, to maintain the consistency of spectral data range, laboratory and field spectral data were resamped and only the spectral band with the same spectrum as the UAV spectrum was retained. Three pretreatment methods, SG + FDR (Savitzky-Golay + First Derivative Reflectance), SG + MSC + FDR (Savitzky-Golay + Multiplicative Scatter Correction + First Derivative Reflectance) and MSC + MF + FDR (Multiplicative Scatter Correction + Median Filter + First Derivative Reflectance), were used to pretreat the three spectra respectively. The pretreatment methods and formulas were shown in Table 1.

**Table 1:** Spectral pretreatment method.

Spectral pretreatment method	Formula	
Savitzky-Golay (SG)	${X}_{i}=\sum_{i=-m}^{i=m}{C}_{j}{X}_{i+j}/N$	${\mathrm{X}}_{\mathrm{i}}$ is the spectral reflectance at wavelength $\mathrm{i}$, ${\mathrm{C}}_{\mathrm{j}}$ is the smoothing coefficient, $\mathrm{N}=2\mathrm{m}+1$ is the size of the smooth window
First derivative reflectance (FDR)	$({R}_{i+\Delta i}-{R}_{i})/\Delta i$	$\mathrm{i}$ is the wavelength, $\Delta \mathrm{i}$ is the wavelength interval, ${\mathrm{R}}_{\mathrm{i}}$ is the spectral reflectance of wavelength $\mathrm{i}$, ${\mathrm{R}}_{\mathrm{i}+\Delta \mathrm{i}}$ is the spectral reflectance of the distance $\Delta \mathrm{i}$ from ${\mathrm{R}}_{\mathrm{i}}$
Multiplicative scatter correction (MSC)	${R}_{i}={m}_{i}\overline{{R }_{i}}+{b}_{i}$ ${R}_{i, MSC}=\frac{\left({R}_{i}-{b}_{i}\right)}{{m}_{i}}$	$\overline{{\mathrm{R} }_{\mathrm{i}}}$ as the standard spectra, $\mathrm{i}$ = 1, 2,…,$\mathrm{n}$, $\mathrm{n}$ is the sample number, ${R}_{i, MSC}$ for the case of a sample of the multiple scattering correction results
Median filter (MF)	$$y = Med\left( {X_{1} ,X_{2} , \ldots ,X_{n} } \right) = \left\{ {\begin{array}{*{20}c} {X_{{i\left( {n + 1} \right)/2}} ,} & {n\,is\,odd} \\ {\frac{1}{2}X_{{i\left( \frac{n}{2} \right)}} + X_{{i\left( {\frac{n}{2} + 1} \right)}} ,} & {n\,is\,even} \\ \end{array} } \right.$$	$\mathrm{y}$ is the median of the sequence, $n$ is the window of the sequence

### Soil moisture factor removed

Direct spectrum conversion (DS) can characterize the influence of soil moisture on the spectrum of UAV through measuring the change law between laboratory spectrum and UAV spectrum. DS conversion has a relationship as shown in Eq. (1).

1 $${X}_{L(m\times p)}={X}_{U(m\times p)}B+k{d}_{s}^{t}$$

where ${X}_{L(m\times p)}$ is the laboratory spectral data of the converted samples, $m$ is the number of converted samples, the Kennard–Stone method is used to select the most appropriate number of converted samples, and $p$ is the number of spectral bands. ${X}_{U(m\times p)}$ is the UAV spectral data of the converted sample, $B$ is the transformation matrix that measures the difference between ${X}_{L(m\times p)}$ and ${X}_{U(m\times p)}$; $k{d}_{s}^{t}$ is the residual matrix used to correct the baseline deviation caused by the difference between the UAV spectrum and the laboratory spectrum. This was the calibration of the UAV spectrum using the laboratory spectrum. If the UAV spectrum needs to be corrected by the field in situ spectrum, the difference between the field in situ spectrum and the UAV spectrum is analyzed, and a functional relationship is established to realize the spectral correction.

An optimal spectral correction method was determined to improve the accuracy of the UAV spectral modeling. In this study, field in-situ spectroscopy was used for calibration, field in-situ spectroscopy, laboratory spectroscopy for superposition calibration, and laboratory spectroscopy for calibration. The modeling accuracies of these three correction methods were compared, and the most suitable correction method was used for subsequent analyses.

### Model construction and evaluation index

Linear and principal component analysis methods were used to eliminate content and spectral outliers of the samples. Seventeen samples were excluded, leaving 279 samples. The concentration gradient method was used to rank the texture content from low to high, and the modeling (186) and verification (93) sets of samples were determined in a 2:1 ratio. PLSR (Partial least-squares regression), RF (Random forest) and XGBoost (extreme Gradient Boosting) were used for modeling. PLSR, which is a classical modelling method, can be used to conduct a detailed analysis of spectral data. The key to this model is determining the optimal number of principal components in the regression model, which is determined using the leave-one cross-validation method. The PLSR algorithm is implemented in the *R* software.. RF can make effective decisions by establishing a regression tree, which has high stability, strong antinoise ability, and strong data adaptability, and cannot easily produce overfitting. The RF algorithm is implemented in the* R* software. XGBoost is an integrated learning model based on decision-tree learning that can deal with nonlinear problems and has high training efficiency. The model can be explained well by ranking the importance of the features. The XGBoost algorithm is implemented in *Python* software.

The accuracy of each model was evaluated using ${R}^{2}$, root-mean-square error (RMSE), relative analysis error (RPD) and quartile error (RPIQ). The closer ${R}^{2}$ is to 1, the higher the accuracy of the model. The closer the RMSE is to 0, the stronger the prediction ability of the model. If RPD < 1.4, the model has no estimation ability. If 1.4 ≤ RPD < 2, the model can perform approximate estimation. If RPD ≥ 2, the model can perform more accurate estimation. If RPIQ < 1.7, the model cannot predict the samples. If 1.7 ≤ RPIQ < 2.5, the model can perform an approximate estimation. If RPIQ ≥ 2.5, the model has a strong prediction ability.

## Results

### Statistical analysis of soil texture content

**Figure 4:**
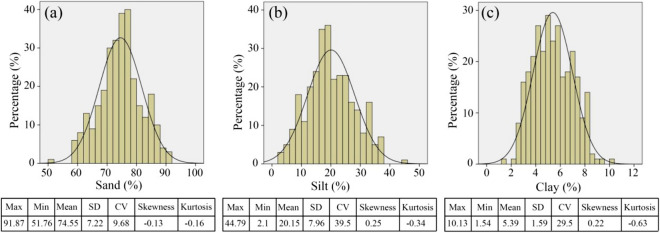
Descriptive statistics of soil texture. SD: standard deviation, CV: coefficient of variation.

The results of the texture content determination of the soil samples are shown in Fig. 4. The contents of sand, silt, and clay ranged from 97.87 to 51.76%, 2.1 to 44.79% and 1.54 to 10.13%, respectively, with an average content of 74.55%, 20.15%, and 5.39%. This indicated that the soil texture in the study area was mainly loam. The standard deviation and coefficient of variation of sand, silt, and clay ranged from 1.59 to 7.96 and 9.68 to 39.5%, representing medium variation. The skewness and kurtosis ranged from − 0.13 to 0.25 and from − 0.63 to − 0.16, indicating that the soil texture content in the study area met the requirement of normal distribution.

### Spectral characteristics and pretreatment

**Figure 5:**
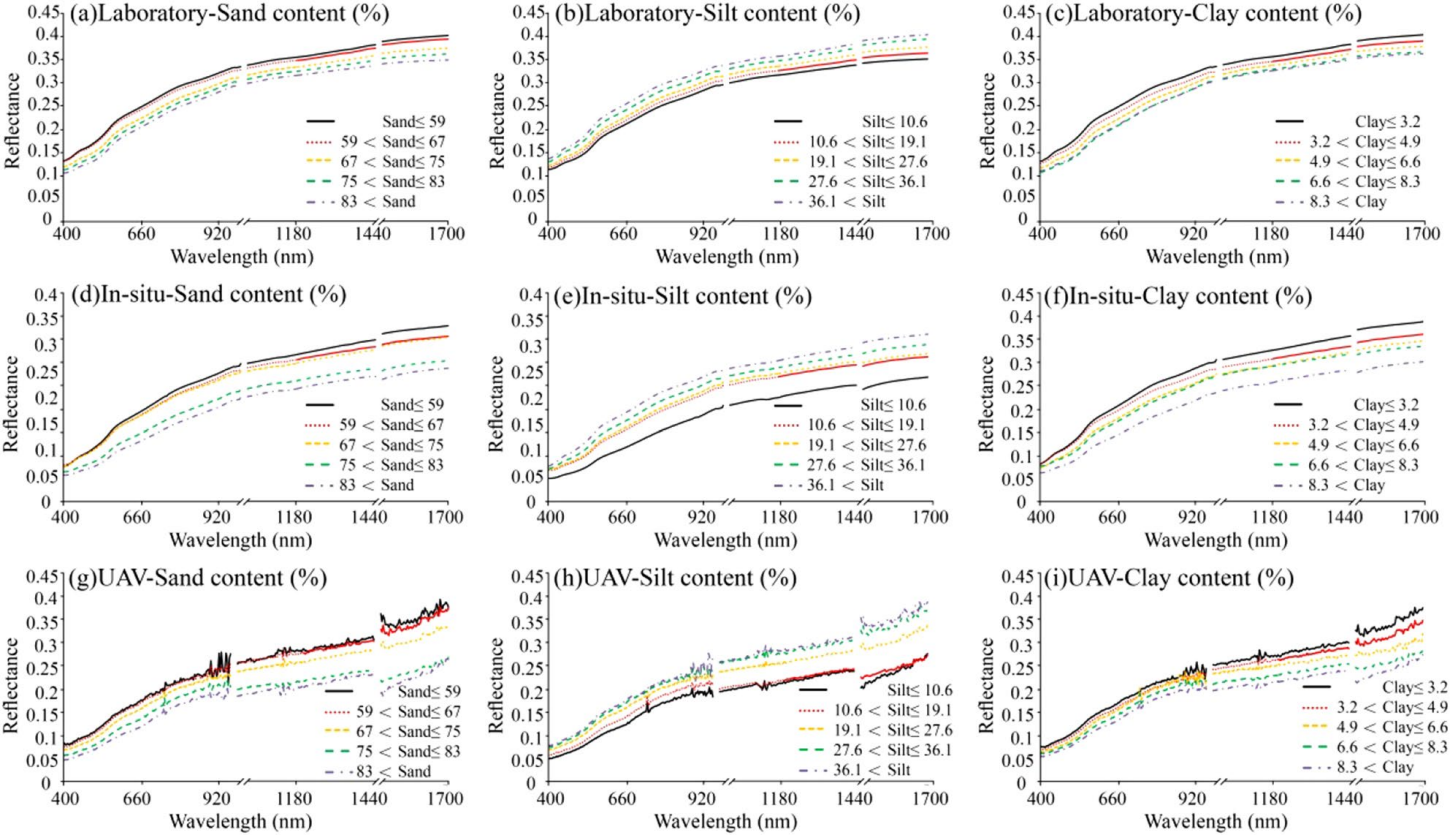
Reflection spectral characteristics of different soil texture contents.

Figure 5 shows the sand, silt and clay contents of all the soil samples divided into five grades in ascending order. The reflectance curves of the laboratory spectrum, field in-situ spectrum, and UAV spectrum were obtained according to the average reflectance of each grade. The overall trends of the three spectral curves for the different soil texture contents were consistent. The spectral reflectance decreases with an increase in sand and clay content, showing a negative correlation, and increases with an increase in silt content, showing a positive correlation. The reflectance value of the laboratory spectrum was higher than that of the field in situ spectrum and the overall UAV spectrum. The three spectra exhibited the same trend, and the reflectance value increased with increasing wavelength.

**Figure 6:**
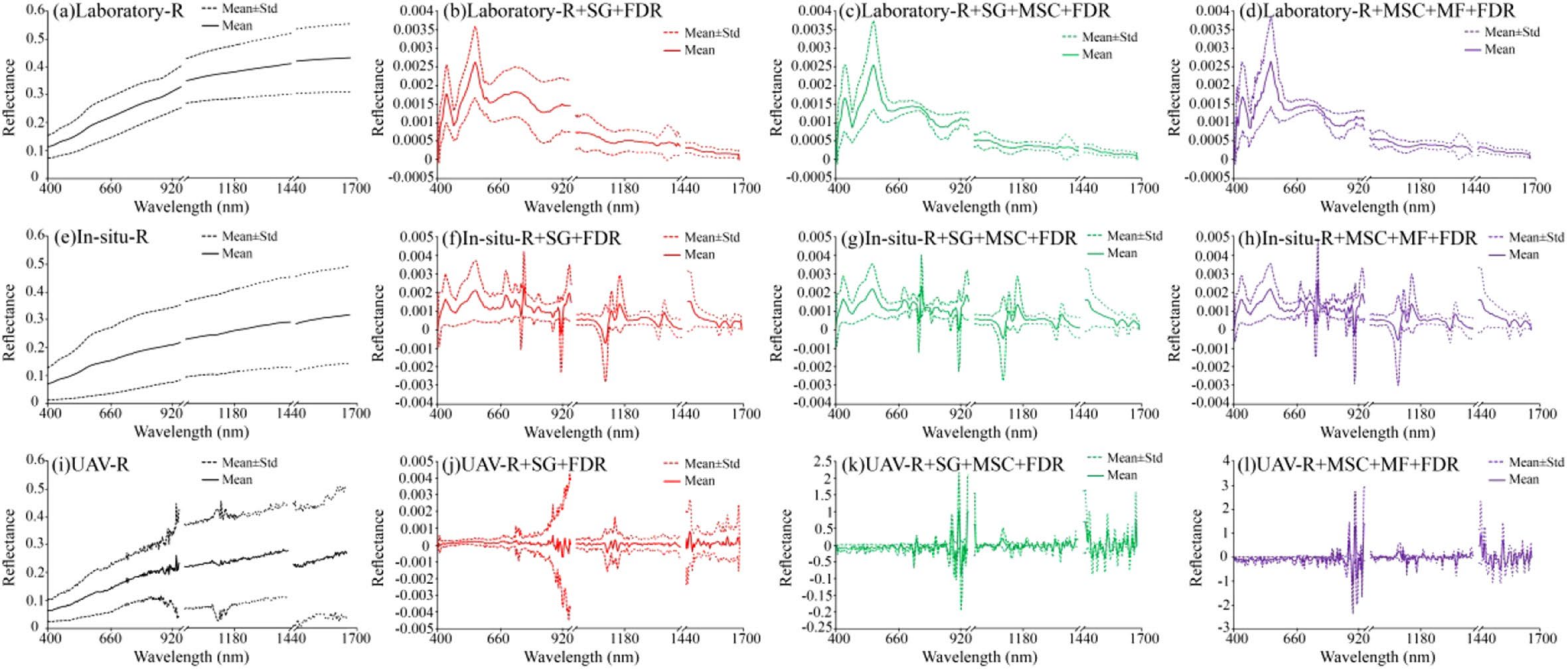
Original spectrum and spectral characteristics after pretreatment.

The pretreated laboratory spectrum, field in-situ spectrum, and UAV spectrum curves are shown in Fig. 6. After the SG + FDR transformation of the three original spectra R, the reflectance of the spectral curves changes, the reflectance value decreases, the spectral characteristics are enhanced, and the number of absorption peaks increases. This indicates that the SG + FDR transformation can highlight the detailed characteristics of the spectral curves. After the SG + MSC + FDR transformation of the original spectrum R (Reflectance), the spectral curve was relatively concentrated. The absorption peak was more prominent than that of the original spectrum R. After the MSC + MF + FDR transformation of the original spectrum R, the spectral cross phenomenon appears in the near-infrared band, indicating that this pretreatment method weakens the grade difference of the spectral curves, makes the spectral features more prominent, amplifies the difference between the peaks and valleys of the spectral curve, and increases the difference in the width of the absorption peak.

### Correlation analysis and extraction of characteristic bands

**Figure 7:**
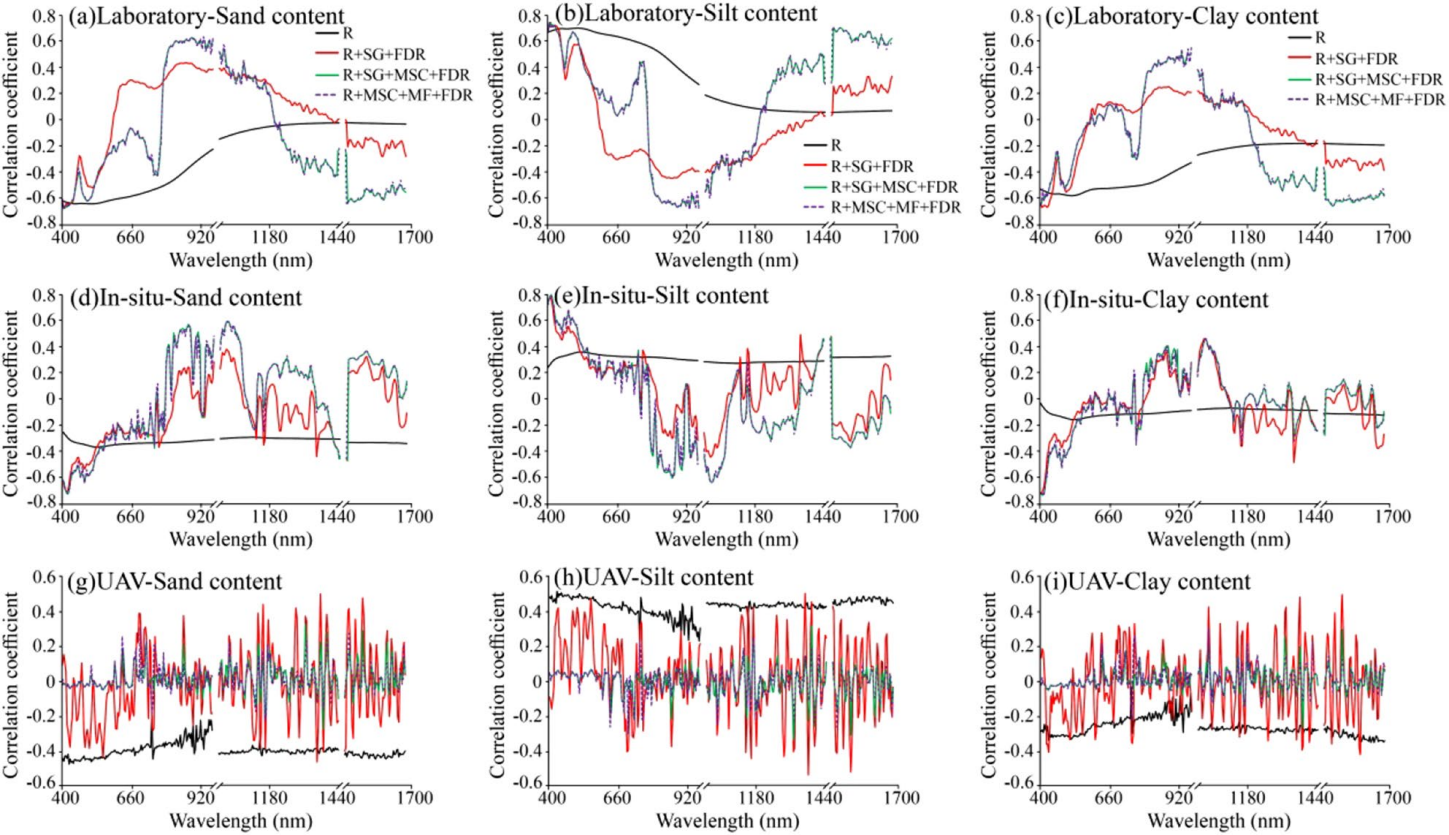
Hyperspectral correlation with soil texture.

A correlation analysis between the laboratory spectrum, field in-situ spectrum, UAV spectrum, and soil texture content was conducted (Fig. 7). The correlation fluctuation of the laboratory spectrum was the smallest, followed by the field in situ and UAV spectra. The correlation between the laboratory spectrum and field in situ spectrum and sand and clay content generally first increased and then decreased, and the correlation with silt content first decreased and then increased. The correlation between the UAV spectrum and soil texture fluctuated at approximately 0.

**Figure 8:**
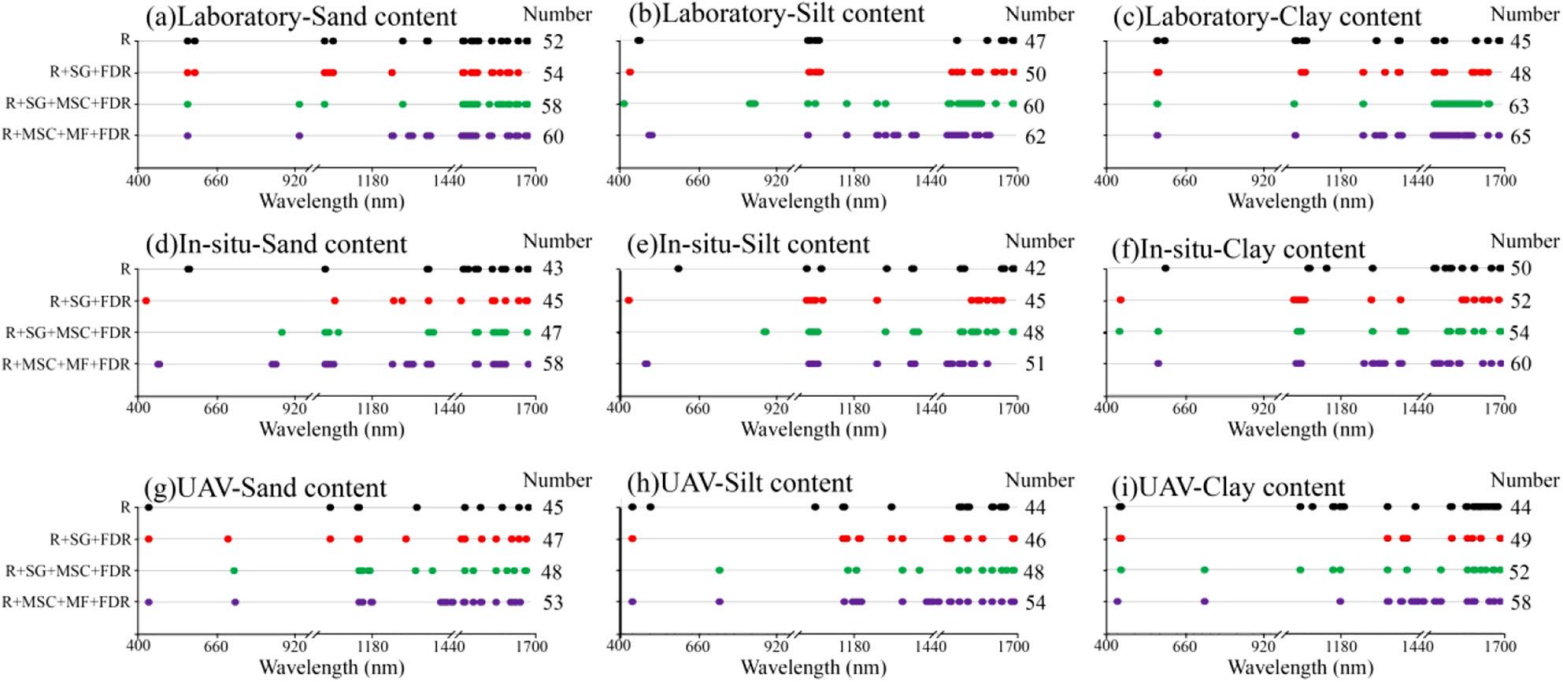
Feature band distribution map.

Characteristic bands corresponding to sand, silt, and clay content were extracted from the laboratory, field in situ, and UAV spectra, respectively (Fig. 8). Overall, the characteristic bands of the three spectra were mainly concentrated in the NIR band (1000–1700 nm), and the characteristic bands in the visible band (400–1000 nm) were lower. Among the four spectral transformation methods, the number of characteristic bands selected by R + MSC + MF + FDR was the largest, followed by R + SG + MSC + FDR and R + SG + FDR. The number of characteristic bands selected by original spectrum R was the least, ranging from 42 to 65.

### Analysis of modeling results of soil texture content

PLSR, RF, and XGBoost modeling with soil texture content was conducted using the laboratory spectrum, field in-situ spectrum, and UAV spectrum, and the modeling results are shown in Tables 2, 3 and 4. In Tables 2, 3 and 4, there is the same pattern of change. For laboratory spectroscopy, PLSR is almost incapable of predicting Sand, RF has the ability to roughly estimate Sand, and XGBoost has a better ability to estimate Sand. In the field in situ spectra, PLSR cannot be estimated by any of the transformations except for R + MSC + MF + FDR which can roughly estimate Sand (RPD = 1.416). In UAV spectroscopy, the overall accuracy of the three modeling methods decreases again, with neither PLSR nor RF having estimation capabilities and XGBoost having only partially better results.

**Table 2:** Sand modeling results.

Spectrum types	Modeling method		Transform method	Calibration			Validation		
				${R}_{cal}^{2}$	${RMSE}_{cal}$	${R}_{val}^{2}$	${RMSE}_{val}$	RPD	RPIQ
Laboratory	PLSR	PC:8	R	0.508	6.014	0.501	6.16	1.155	1.351
		PC:8	R + SG + FDR	0.526	5.516	0.507	5.71	1.246	1.457
		PC:9	R + SG + MSC + FDR	0.548	5.033	0.501	5.274	1.349	1.578
		PC:8	R + MSC + MF + FDR	**0.552**	**5.009**	**0.537**	**5.133**	**1.386**	**1.621**
	RF		R	0.587	4.806	0.566	4.883	1.457	1.704
			R + SG + FDR	0.607	4.669	0.598	4.772	1.491	1.744
			R + SG + MSC + FDR	0.618	4.621	0.601	4.75	1.498	1.752
			R + MSC + MF + FDR	**0.624**	**4.519**	**0.618**	**4.724**	**1.506**	**1.761**
	XGBoost		R	0.621	4.563	0.601	4.737	1.502	1.757
			R + SG + FDR	0.639	4.019	0.609	4.289	1.659	1.94
			R + SG + MSC + FDR	0.655	3.788	0.639	3.988	1.784	2.087
			R + MSC + MF + FDR	**0.661**	**3.691**	**0.647**	**3.813**	**1.866**	**2.182**
In-situ	PLSR	PC:7	R	0.517	5.633	0.499	5.842	1.218	1.424
		PC:8	R + SG + FDR	0.529	5.445	0.507	5.651	1.259	1.472
		PC:9	R + SG + MSC + FDR	0.555	4.811	0.537	5.097	1.396	1.633
		PC:8	R + MSC + MF + FDR	**0.569**	**4.917**	**0.557**	**5.025**	**1.416**	**1.656**
	RF		R	0.528	5.463	0.507	5.697	1.249	1.461
			R + SG + FDR	0.543	4.985	0.522	5.282	1.347	1.575
			R + SG + MSC + FDR	0.577	4.772	0.566	4.944	1.439	1.683
			R + MSC + MF + FDR	**0.589**	**4.553**	**0.561**	**4.87**	**1.461**	**1.709**
	XGBoost		R	0.594	4.593	0.587	4.788	1.486	1.738
			R + SG + FDR	0.603	4.628	0.588	4.766	1.493	1.746
			R + SG + MSC + FDR	0.615	4.519	0.601	4.75	1.498	1.752
			R + MSC + MF + FDR	**0.627**	**4.444**	**0.604**	**4.663**	**1.526**	**1.784**
UAV	PLSR	PC:6	R	0.487	6.769	0.453	6.941	1.025	1.199
		PC:8	R + SG + FDR	0.503	6.071	0.483	6.15	1.157	1.353
		PC:7	R + SG + MSC + FDR	0.519	5.598	0.501	5.803	1.226	1.434
		PC:8	R + MSC + MF + FDR	**0.522**	**5.487**	**0.507**	**5.607**	**1.269**	**1.484**
	RF		R	0.497	5.987	0.453	6.144	1.158	1.354
			R + SG + FDR	0.518	5.502	0.501	5.78	1.231	1.44
			R + SG + MSC + FDR	0.537	5.197	0.507	5.49	1.296	1.516
			R + MSC + MF + FDR	**0.559**	**5.176**	**0.524**	**5.386**	**1.321**	**1.545**
	XGBoost		R	0.533	5.169	0.501	5.477	1.299	1.519
			R + SG + FDR	0.559	5.147	0.513	5.354	1.329	1.554
			R + SG + MSC + FDR	0.577	4.653	0.549	4.972	1.431	1.674
			R + MSC + MF + FDR	**0.586**	**4.596**	**0.571**	**4.847**	**1.468**	**1.717**

Bold is the value with the highest accuracy in each model.

**Table 3:** Silt modeling results.

Spectrum types	Modeling method		Transform method	Calibration			Validation		
				${R}_{cal}^{2}$	${RMSE}_{cal}$	${R}_{val}^{2}$	${RMSE}_{val}$	RPD	RPIQ
Laboratory	PLSR	PC:7	R	0.573	5.326	0.547	5.535	1.418	1.918
		PC:8	R + SG + FDR	0.586	5.024	0.551	5.394	1.455	1.968
		PC:8	R + SG + MSC + FDR	0.608	5.011	0.583	5.335	1.471	1.99
		PC:8	R + MSC + MF + FDR	**0.621**	**4.989**	**0.607**	**5.281**	**1.486**	**2.011**
	RF		R	0.604	5.122	0.583	5.342	1.469	1.988
			R + SG + FDR	0.639	5.147	0.621	5.372	1.461	1.977
			R + SG + MSC + FDR	0.651	4.693	0.632	4.745	1.654	2.238
			R + MSC + MF + FDR	**0.669**	**4.521**	**0.642**	**4.680**	**1.677**	**2.269**
	XGBoost		R	0.661	4.369	0.631	4.697	1.671	2.261
			R + SG + FDR	0.689	4.317	0.655	4.579	1.714	2.319
			R + SG + MSC + FDR	0.701	3.662	0.672	3.832	2.048	2.771
			R + MSC + MF + FDR	**0.713**	**3.455**	**0.682**	**3.695**	**2.124**	**2.874**
In-situ	PLSR	PC:8	R	0.541	5.887	0.503	6.088	1.289	1.744
		PC:8	R + SG + FDR	0.559	5.611	0.523	5.865	1.338	1.81
		PC:9	R + SG + MSC + FDR	0.579	5.302	0.554	5.531	1.419	1.92
		PC:8	R + MSC + MF + FDR	**0.581**	**5.143**	**0.573**	**5.39**	**1.456**	**1.97**
	RF		R	0.572	5.229	0.544	5.519	1.422	1.924
			R + SG + FDR	0.596	5.143	0.567	5.346	1.468	1.986
			R + SG + MSC + FDR	0.617	4.995	0.597	5.156	1.522	2.059
			R + MSC + MF + FDR	**0.621**	**4.988**	**0.607**	**5.123**	**1.532**	**2.073**
	XGBoost		R	0.603	5.06	0.577	5.215	1.505	2.036
			R + SG + FDR	0.622	4.778	0.599	4.945	1.587	2.147
			R + SG + MSC + FDR	0.644	4.663	0.608	4.896	1.603	2.169
			R + MSC + MF + FDR	**0.658**	**4.124**	**0.637**	**4.387**	**1.789**	**2.42**
UAV	PLSR	PC:7	R	0.498	7.014	0.453	7.213	1.088	1.472
		PC:10	R + SG + FDR	0.518	6.223	0.498	6.412	1.224	1.656
		PC:8	R + SG + MSC + FDR	0.539	5.775	0.514	5.91	1.328	1.797
		PC:8	R + MSC + MF + FDR	**0.541**	**5.663**	**0.522**	**5.879**	**1.335**	**1.806**
	RF		R	0.481	7.649	0.476	7.809	1.005	1.36
			R + SG + FDR	0.542	5.802	0.501	6.042	1.299	1.757
			R + SG + MSC + FDR	0.561	5.323	0.533	5.554	1.413	1.912
			R + MSC + MF + FDR	**0.571**	**5.221**	**0.559**	**5.507**	**1.425**	**1.928**
	XGBoost		R	0.539	5.617	0.517	5.874	1.336	1.808
			R + SG + FDR	0.569	5.336	0.542	5.562	1.411	1.909
			R + SG + MSC + FDR	0.587	5.213	0.571	5.435	1.444	1.954
			R + MSC + MF + FDR	**0.594**	**5.255**	**0.581**	**5.35**	**1.467**	**1.985**

Bold is the value with the highest accuracy in each model.

**Table 4:** Clay modeling results.

Spectrum types	Modeling method		Transform method	Calibration			Validation		
				${R}_{cal}^{2}$	${RMSE}_{cal}$	${R}_{val}^{2}$	${RMSE}_{val}$	RPD	RPIQ
Laboratory	PLSR	PC:8	R	0.652	0.816	0.617	0.902	1.779	2.676
		PC:9	R + SG + FDR	0.671	0.748	0.649	0.885	1.814	2.727
		PC:8	R + SG + MSC + FDR	0.695	0.719	0.669	0.834	1.924	2.894
		PC:8	R + MSC + MF + FDR	**0.705**	**0.663**	**0.684**	**0.75**	**2.141**	**3.218**
	RF		R	0.686	0.744	0.666	0.868	1.848	2.781
			R + SG + FDR	0.713	0.603	0.698	0.714	2.247	3.38
			R + SG + MSC + FDR	0.738	0.519	0.714	0.683	2.351	3.534
			R + MSC + MF + FDR	**0.759**	**0.511**	**0.725**	**0.656**	**2.448**	**3.679**
	XGBoost		R	0.736	0.596	0.701	0.684	2.346	3.529
			R + SG + FDR	0.771	0.603	0.748	0.642	2.501	3.759
			R + SG + MSC + FDR	0.793	0.598	0.768	0.625	2.567	3.862
			R + MSC + MF + FDR	**0.801**	**0.579**	**0.786**	**0.601**	**2.669**	**4.016**
In-situ	PLSR	PC:10	R	0.619	0.983	0.593	1.046	1.534	2.307
		PC:6	R + SG + FDR	0.647	0.907	0.619	0.951	1.687	2.538
		PC:8	R + SG + MSC + FDR	0.662	0.887	0.633	0.966	1.661	2.498
		PC:8	R + MSC + MF + FDR	**0.678**	**0.836**	**0.659**	**0.937**	**1.712**	**2.576**
	RF		R	0.659	0.849	0.617	0.996	1.612	2.423
			R + SG + FDR	0.682	0.716	0.659	0.875	1.834	2.758
			R + SG + MSC + FDR	0.702	0.696	0.672	0.769	2.086	3.138
			R + MSC + MF + FDR	**0.719**	**0.618**	**0.698**	**0.712**	**2.255**	**3.39**
	XGBoost		R	0.688	0.773	0.659	0.863	1.859	2.797
			R + SG + FDR	0.709	0.634	0.679	0.741	2.167	3.257
			R + SG + MSC + FDR	0.731	0.598	0.716	0.681	2.357	3.544
			R + MSC + MF + FDR	**0.749**	**0.512**	**0.724**	**0.66**	**2.431**	**3.657**
UAV	PLSR	PC:7	R	0.501	1.293	0.472	1.428	1.124	1.69
		PC:8	R + SG + FDR	0.522	1.058	0.498	1.266	1.268	1.906
		PC:8	R + SG + MSC + FDR	0.543	1.093	0.529	1.177	1.364	2.051
		PC:8	R + MSC + MF + FDR	**0.551**	**1.002**	**0.539**	**1.149**	**1.397**	**2.101**
	RF		R	0.531	1.079	0.506	1.156	1.388	2.088
			R + SG + FDR	0.556	1.025	0.514	1.151	1.394	2.097
			R + SG + MSC + FDR	0.572	1.102	0.552	1.121	1.432	2.153
			R + MSC + MF + FDR	**0.589**	**0.987**	**0.671**	**1.08**	**1.486**	**2.235**
	XGBoost		R	0.579	1.033	0.541	1.109	1.447	2.176
			R + SG + FDR	0.605	0.983	0.584	1.081	1.484	2.233
			R + SG + MSC + FDR	0.614	0.956	0.593	1.061	1.512	2.275
			R + MSC + MF + FDR	**0.629**	**0.984**	**0.601**	**1.023**	**1.569**	**2.359**

Bold is the value with the highest accuracy in each model.

Among the three spectral modeling results, the modeling accuracy of the laboratory spectrum was the highest, followed by that of the field in situ spectrum, and the modeling accuracy of the UAV spectrum was the lowest. Among the three modeling methods, XGBoost had the highest modeling accuracy, followed by RF, while PLSR had the lowest. Among the four transformation methods, the modeling accuracy of R + MSC + MF + FDR is the highest overall, followed by R + SG + MSC + FDR and R + SG + FDR, and the modeling accuracy of original spectrum R is the lowest. Among the modeling results for soil sand, silt, and clay content, clay had the highest overall modeling accuracy, followed by silt and sand.

### UAV spectral correction

**Figure 9:**
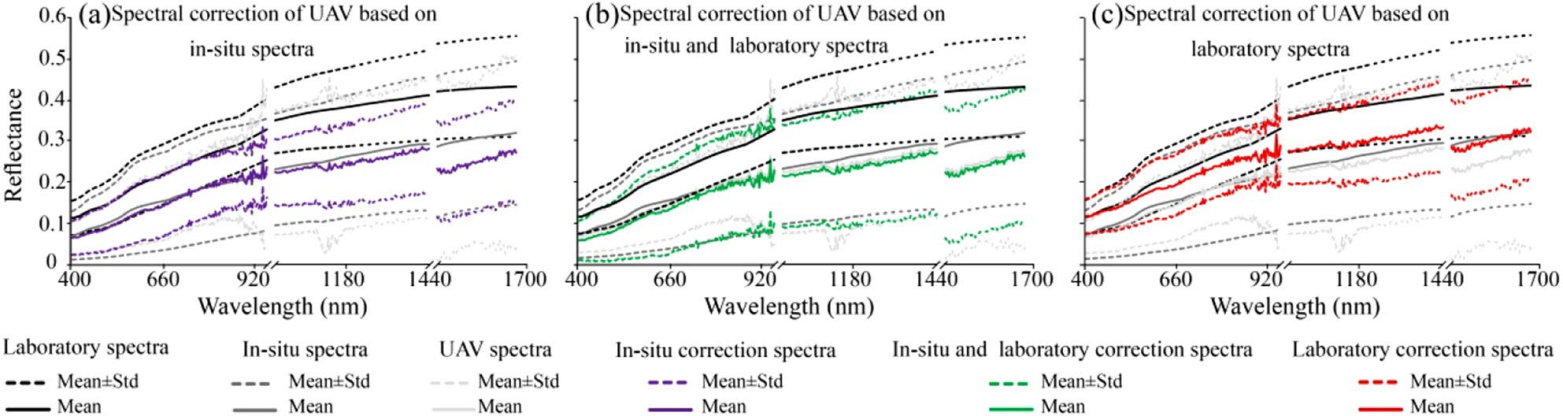
Comparison of UAV spectral correction.

Field in-situ spectra and laboratory spectra were used as reference spectra to calibrate the UAV spectrum, and the corrected results are shown in Fig. 9. The corrected spectrum in figure (a) is similar to the field in situ spectrum, but the spectral reflectance is lower than the laboratory spectrum. (b) The difference between the maximum and minimum values of the corrected spectrum increased, which magnified the differential characteristics of the spectral curve, indicating that this is a cumulative error caused by the superposition correction. (c) The corrected spectrum in the figure is similar to the laboratory spectrum, and the reflectance difference between the maximum and minimum spectral values was reduced, indicating that the laboratory spectrum has the strongest correction effect on the UAV spectrum.

**Figure 10:**
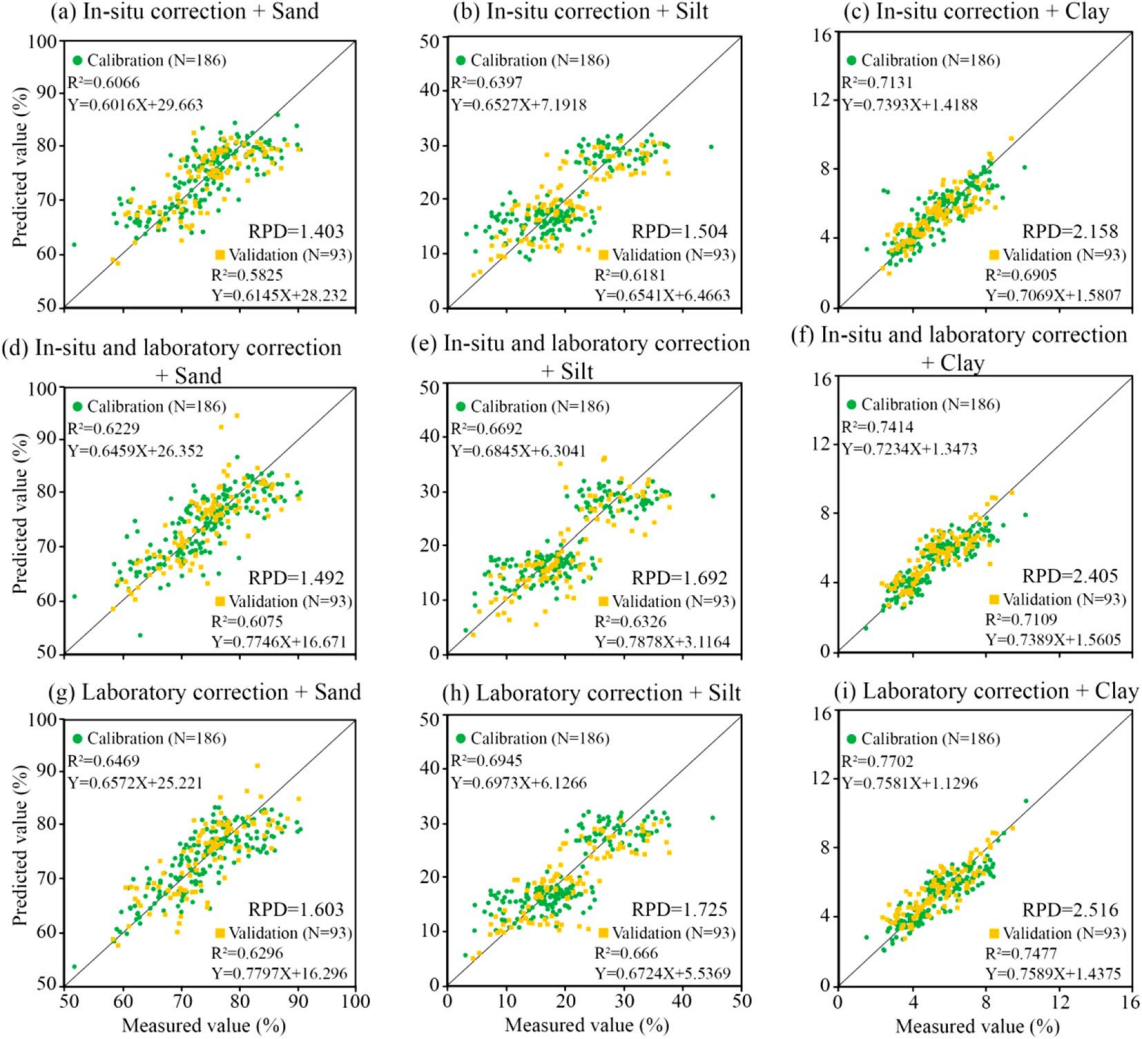
Spectral modeling scatter plot after each correction.

To further verify the correction effects of these three methods, the three types of corrected spectra were pretreated with R + MSC + MF + FDR, and the silt and clay contents were individually subjected to XGBoost modeling. The modeling results are shown in Fig. 10. The overall accuracy of the laboratory spectrum correction was the highest, the accuracy of the field in situ spectrum and laboratory spectrum superposition correction was second, and the accuracy of the field in situ spectrum correction was the lowest.

### Analysis of cartographic results of soil texture content

**Figure 11:**
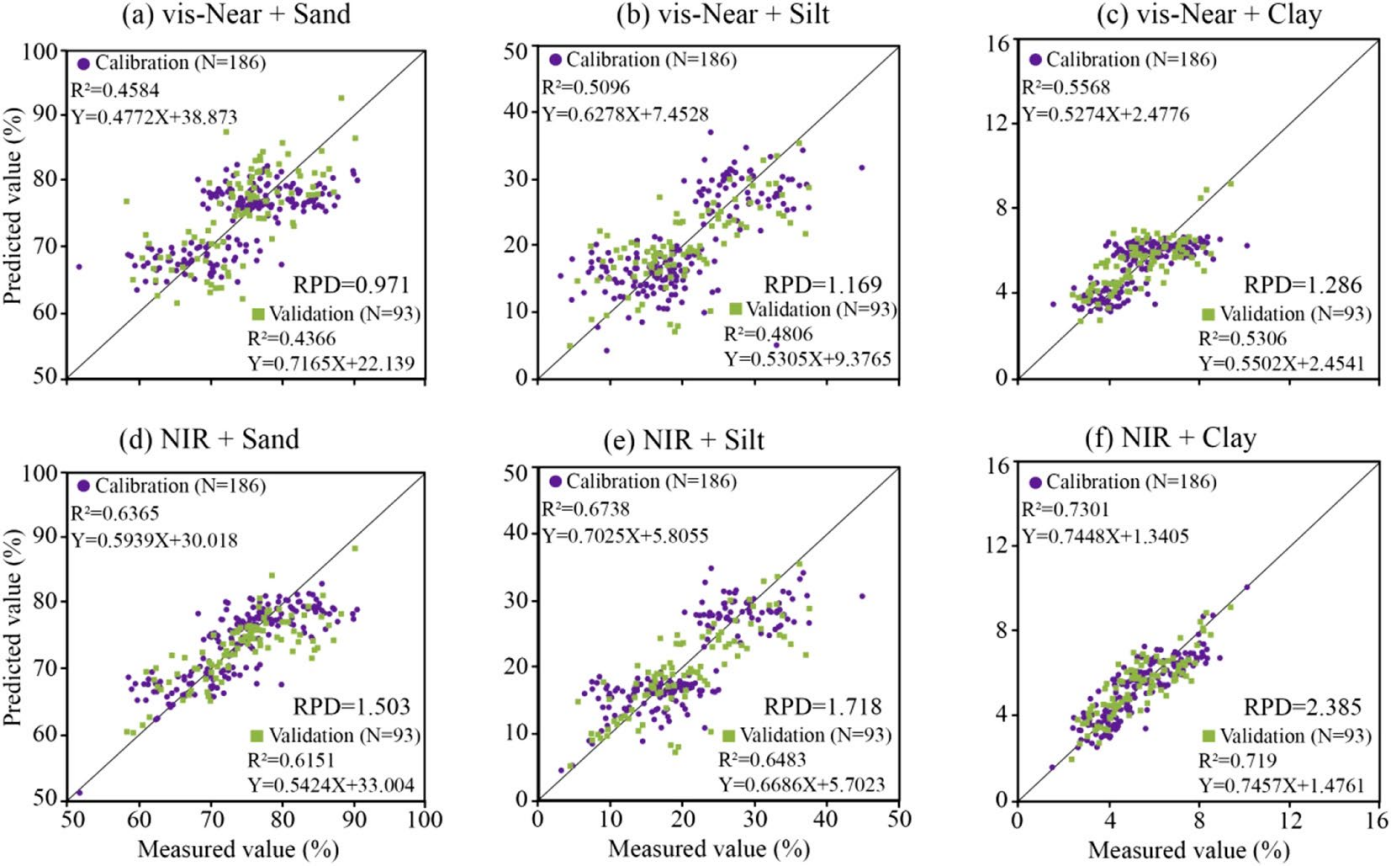
Modeling scatter plot of vis-Near and NIR spectra.

Given that the model established with a 400–1700 nm spectrum cannot be used for a single-phase mechanism diagram, R + MSC + MF + FDR was separately preprocessed for spectral data of the visible-near camera and NIR camera, and after correction with laboratory spectrum, it was used for XGBoost modeling with sand, silt, and clay content one by one. The modeling results are shown in Fig. 11. The models established by vis-Near with sand, silt, and clay content were not capable of estimation (RPD < 1.4). The models established using NIR and sand, silt, and clay content can be estimated (RPD > 1.4).

Soil texture (Sand, Silt and Clay) was mapped in this study as follows. First, the actual sampling points in the field were imported into the UAV hyperspectral images and the corresponding UAV hyperspectral data for each sampling point were extracted. Second, XGBoost modeling was performed based on UAV hyperspectral data to establish regression equations for Soil texture (Sand, Silt and Clay) versus spectral bands for each farmland zone. Finally, Soil texture (Sand, Silt and Clay) mapping was performed by substituting the regression equations into the UAV hyperspectral images in the band calculator of the ENVI software. In Soil texture (Sand, Silt and Clay) mapping, the geostatistical models used for the three components are all spherical models.

**Figure 12:**
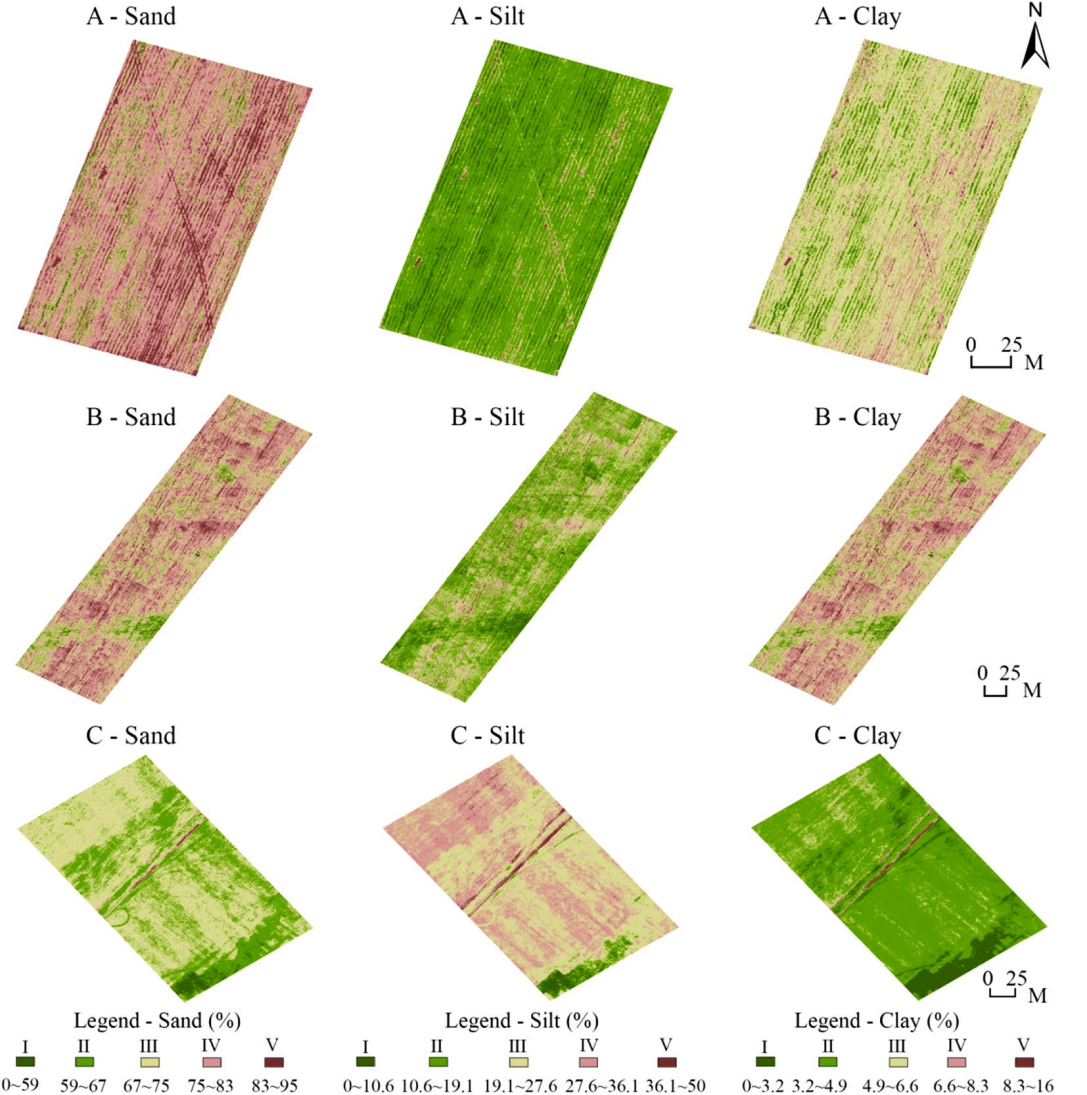
Soil texture mapping results.

In the ENVI software, the model formula established in the previous step was substituted into the hyperspectral images captured by the NIR camera to perform sand, silt, and clay content mapping, and the mapping results of the three farmland areas were obtained (Fig. 12). In the sand content mapping results, Grade IV was the main sand content in Farmlands A and B, and Grade III was the main sand content in Farmland C. In the cartographic results of silt content, Field Districts A and B mainly had Grade II, whereas Field District C mainly had Grade IV. In the mapping results, the clay content was mainly Grade III in Farmland Areas A, III, and IV in Farmland Area B, and Grade II in Farmland Area C.

Content statistics were performed on the mapping results to obtain the area and percentage of each grade and the average content value of each farmland area (Table 5). In the statistics for sand content, the average sand content in Farmland A was the highest (80.15%), followed by that of Farmland B (77.03%) and that of Farmland C (67.71%). In the silt content statistics, the average content of Field C was the highest (28.47%), followed by Field B (16.64%) and Field A has the lowest (13.79%). In the statistics for clay content, the average clay content in Farmland B is the highest (6.33%), followed by Farmland A (6.06%) and Farmland C (3.82%).

**Table 5:** Statistical results of texture content.

	I		II		III		IV		V		Mean content
	m^2^	%	m^2^	%	m^2^	%	m^2^	%	m^2^	%	
Sand											
A	118	0.59	450	2.25	1502	7.51	12,068	60.34	5862	29.31	80.15
B	476	1.19	1152	2.88	12,688	31.72	19,160	47.9	6524	16.31	77.03
C	1317	3.92	12,849	38.24	17,788	52.94	1485	4.42	161	0.48	67.71
Silt											
A	6206	31.03	11,724	58.62	1792	8.96	194	0.97	84	0.42	13.79
B	5044	12.61	22,856	57.14	11,332	28.33	504	1.26	264	0.66	16.64
C	998	2.97	1381	4.11	16,141	48.04	14,166	42.16	914	2.72	28.47
Clay											
A	298	1.49	4172	20.86	13,104	65.52	2082	10.41	344	1.72	6.06
B	1620	4.05	4432	11.08	14,788	36.97	16,472	41.18	2688	6.72	6.33
C	7227	21.51	22,730	67.65	2255	6.71	833	2.48	554	1.65	3.82

## Discussion

Based on four spectral transforms, three modeling methods, and three types of spectra, soil texture inversion models were established for sand, silt, and clay. It was found that appropriate model regression methods based on UAV remote sensing could be used for quantitative inversion of soil texture content, which was consistent with the research of Gholizadeh et al.^31^.

Yang et al.^32^ showed that for spectral modeling and soil mapping, the application of machine learning provided a more ideal spectral modeling scheme, which greatly improved the prediction accuracy and robustness of the estimation model. Ye et al.^33^ indicate that integrated learning, as an important area of machine learning, has received much attention in machine learning and data mining research. There is a lot of soil modeling and mapping represented by deep learning, however, there is less research on soil mapping with integrated learning algorithms^34^. Therefore, soil texture mapping of UAV hyperspectral images using integrated learning is attempted in this study.

The PLSR method had a relatively low ability to deal with nonlinearity and was prone to randomness, which affected the nonlinear relationship between soil texture and spectral reflectance, resulting in a poorer modeling effect than the RF method. This was in line with the results of Chagas et al.^35^ Chagas used the RF and PLSR models to predict the spatial distribution of soil texture in semi-arid areas and found that the RF model had higher accuracy. Compared to the traditional regression model, the XGBoost regression algorithm model introduced in this study has a strong learning ability, ability to approximate most nonlinear relations, strong generalization ability, strong robustness, and fault tolerance, and could handle nonlinear and high-dimensional problems effectively to achieve local optimization. Generally, this was more effective than the traditional regression model^36,37^. Ge et al.^38^ used UAV hyperspectral images to predict agricultural soil moisture content by comparing the XGBoost and RF modeling methods combined with the soil moisture spectral index. The results showed that the XGBoost model had the strongest effect on the soil moisture content estimation and the highest modeling accuracy, with R^2^ of 0.926 and an RPD value of 2.556. This is the same as the optimal modeling method used in this study.

Bilgili et al.^39^ conducted a visible near-infrared spectrum prediction of sand, silt, and clay contents in soil samples from the same soil class in a single field in Turkey. Here, clay content had the highest prediction accuracy (R^2^ = 0.90, RPD = 3.08) and sand content had the lowest accuracy (R^2^ = 0.35, RPD = 1.28). Awiti et al.^40^ used vis–NIR spectral technology to conduct soil attribute prediction and grade classification for tropical soils in sub-Saharan Africa, where the prediction accuracies of sand, silt, and clay content were R^2^ = 0.82, 0.83, and 0.87, respectively. This indicated that clay content had the highest estimation accuracy. Sand content was estimated to have the lowest accuracy, which is consistent with the results of this study.

In order to solve the problem of oscillation during the flight of the drone. In conjunction with Shu et al.^12^ First, a sunny, cloudless day with less than force three winds was chosen for the drone flight. Second, the flight path was checked in time after the flight was completed using the Sbgcenter software. If severe oscillations occurred during the flight, the flight was re-run. Finally, the UAV images are orthorectified to avoid serious spectral errors.

The soil texture inversion model established based on the farmland scale achieved a relatively high level of accuracy, and the inversion results were consistent with the results measured. Compared with Sankey et al.^41^, who studied the soil nutrient content of pasture and farmland in Seville, New Mexico, using Sentinel aerial satellite and UAV hyperspectral images, the UAV data were less affected by atmospheric absorption, scattering, and cloud cover. The flight test can be conducted by artificially selecting a more suitable external environment and the most suitable time for flight to obtain image data with greater imaging quality. This can reflect the real situation of the grade difference in soil texture content in farmlands more clearly and accurately. Therefore, UAV images can successfully estimate the soil nutrient content in farmlands, which is the same as the result of soil texture estimation using the UAV hyperspectral method in this study.

Compared with the spectral data obtained under laboratory conditions, the signal-to-noise ratio of UAV spectral data obtained under natural field light conditions is usually lower owing to the influence of soil moisture in the field. Therefore, when such spectral data are used to estimate the soil texture content, the accuracy of the model needs to be further improved. Compared with the original data, the texture inversion model established using UAV data after soil moisture removal and correction improved the model stability and inversion effect overall. Ji et al.^42^ used a direct conversion method for the spectral correction of paddy soils in Zhejiang Province, and the accuracy of the modeling model was significantly improved. The results have shown that removing water from the soil spectra can improve the prediction accuracy of the soil texture, which is consistent with the results of this study. The combination of corrected UAV data and the introduction of machine learning algorithms effectively improved the accuracy and reliability of the UAV remote sensing inversion model, confirming the necessity of eliminating the corresponding interference background in the field of UAV remote sensing monitoring.

The corrected spectral data are highly comparable and transitive, effectively reducing the influence of the field environment on the field spectrum. With the support of the university’s positive method, future estimation of soil texture content based on hyperspectral data is expected to eliminate the additional cost of soil sample collection caused by the construction of an independent soil texture model, which significantly improves the use efficiency of UAV spectral data and verifies the potential of laboratory model migration for field applications. This study has provided a technical reference for the wide application of UAV hyperspectral data in soil environmental monitoring, digital soil mapping, precision agriculture, and other fields.

## Conclusion

The applicability of the UAV-compatible high-resolution spectrograph for the rapid estimation of the soil texture content in farmlands was explored. Based on three farmland areas of the Huangshui Watershed in the Qinghai Province, hyperspectral data were collected using a UAV-mounted hyperspectral camera under field conditions, and a soil texture estimation model was constructed. The results show that UAV hyperspectral imagery combined with machine learning can obtain a set of ideal processing methods. The pre-processing of the spectral data can obtain high accuracy of soil texture estimation and good mapping effect. The results of this study can provide effective technical support and decision-making assistance for agricultural land planning on the Tibetan Plateau. The main innovation of this study is to establish a set of processing procedures and methods applicable to UAV hyperspectral imagery to provide data reference for monitoring soil texture in agricultural fields on the Tibetan Plateau.

## Data Availability

The datasets generated and analysed during the current study are not publicly available due fund projects require confidentiality but are available from the corresponding author on reasonable request.
